# Supplementary material for: Hsp70–Bag3 Module Regulates Macrophage Motility and Tumor Infiltration via Transcription Factor LITAF and CSF1
Source: Cancers (Basel). 2022 Aug 28;14(17):4168. doi: 10.3390/cancers14174168 (PMC9454964; doi:10.3390/cancers14174168)
Supplement: Supplementary file 1 [file cancers-14-04168-s001.zip › cancers-1859008-supplementary.pdf]

## Supplement figure legends

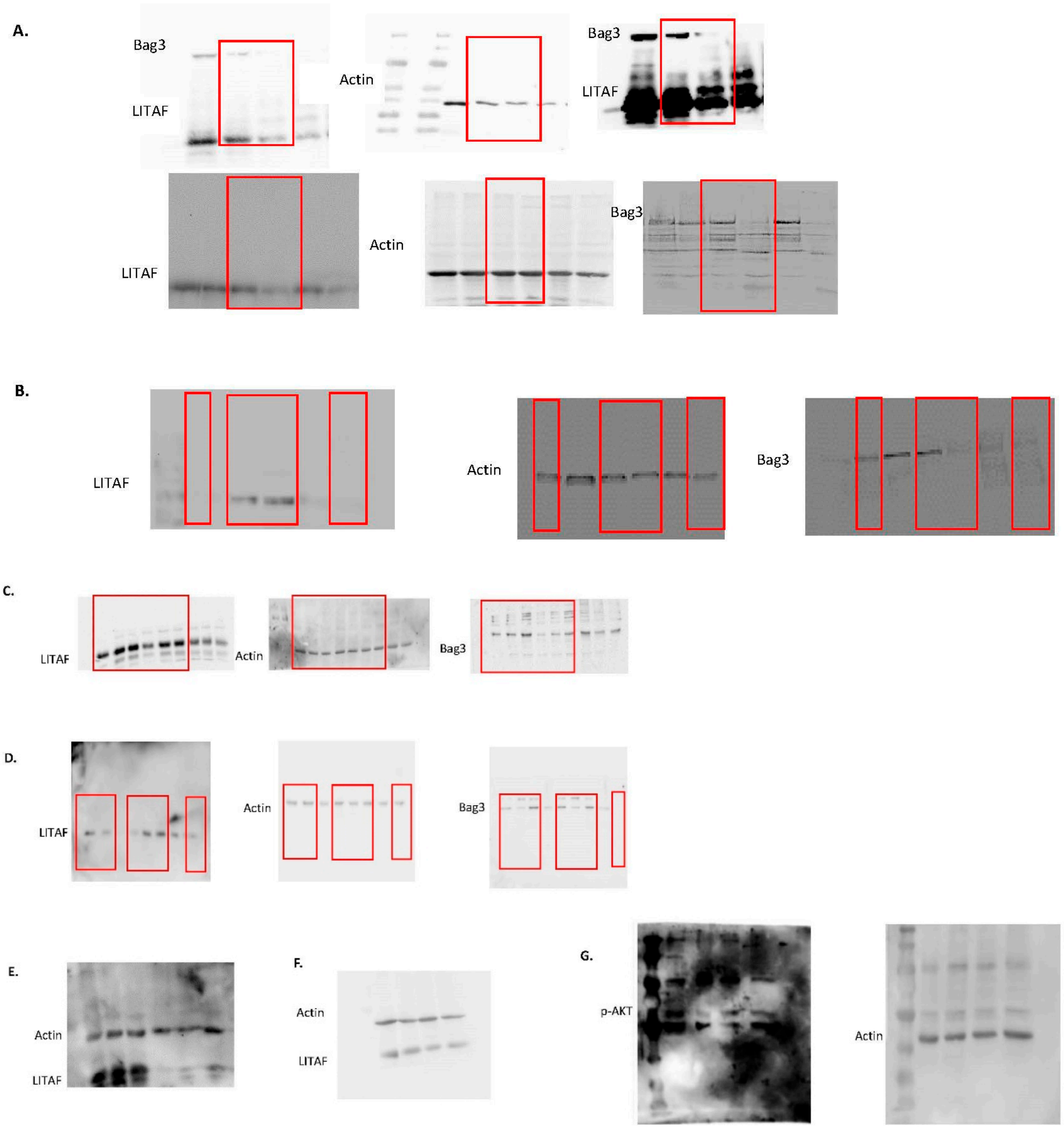


**Figure S1** Corresponding uncropped blots to Figures (A) 1 (B) 3B (C) 3C (D) 3D (E)3F (F) 3F (G) 5C. Red squares represent the bands that were cropped and used in the final figures

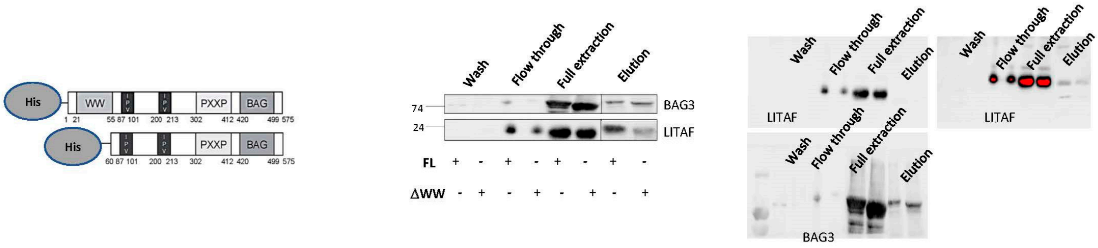


**Figure S2** LITAF binds to BAG3 WW domain. 293T cells were transfected with full length or WW deleted (DWW) BAG3 His-tagged plasmids. Cells were lysed and constructs were pulled down using Hispur Ni-NTA beads. Left upper panel- Full length BAG3 construct. Left lower panel DWW BAG3 construct. Right panel- Western

blot results of the different stages of the pull down with corresponding uncropped blots.

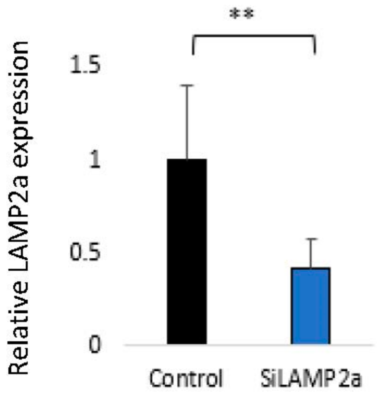


**Figure S3** Relative expression of LAMP2a in control and LAMP2a depleted cells. THP1 cells were transfected with indicated LAMP2a siRNA for 72 h followed by real-time PCR analysis of LAMP2a mRNA levels. Depicited is the mean relative expression (log2) ±s.e.m. **p≤0.01.

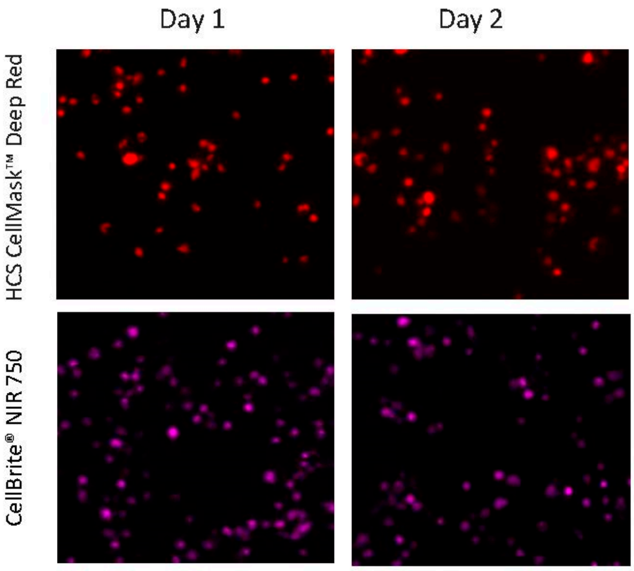


**Figure S4** Cell membrane stain 24h follow up THP1 cells were plated and differentiated with 120nm PMA. The next day cells were stained with CellMask deep Red or CellBrite NIR 750 for 90 minutes. Then cells were washed, and pictures were taken with fluorescent microscope right after wash and 24h later

**Table S1:** Common Upregulated and downregulated genes in Bag3 SiRNA, LITAF SiRNA and JG98 treated THP1 cells according to RNA sequencing data.

| Common_genes_UPREGULATED_1257 |
|---|
| HES4 |
| ISG15 |
| NADK |
| TNFRSF14 |
| THAP3 |
| FBXO6 |
| DHRS3 |
| ARHGEF19 |
| CPLANE2 |
| NBL1 |
| TMCO4 |
| C1QA |
| LYPLA2 |
| HMGCL |
| FUCA1 |
| PNRC2 |
| TMEM50A |
| GPATCH3 |
| LINC02574 |
| IFI6 |
| STX12 |
| LAPTM5 |
| SPOCD1 |
| TMEM234 |
| RNF19B |
| ADPRHL2 |
| EVA1B |
| C1orf122 |
| RRAGC |
| LOC105378663 |
| RLF |
| ZNF684 |
| RIMKLA |
| TESK2 |
| HSPB11 |
| TMEM59 |
| PDE4B |
| TCTEX1D1 |
| WDR78 |
| MIER1 |
| IL23R |

| Common_genes_DOWNREGULATED_1504 |
|---|
| LINC01128 |
| KLHL17 |
| ACAP3 |
| FNDC10 |
| SLC35E2B |
| SLC35E2A |
| SKI |
| ARHGEF16 |
| TP73 |
| NPHP4 |
| GPR153 |
| PLEKHG5 |
| NOL9 |
| DNAJC11 |
| PER3 |
| ERRFI1 |
| SLC45A1 |
| RERE |
| TMEM201 |
| PIK3CD |
| UBE4B |
| DFFA |
| SRM |
| EXOSC10 |
| MTOR |
| UBIAD1 |
| KAZN |
| ZBTB17 |
| PADI2 |
| RCC2 |
| CAMK2N1 |
| CDA |
| HP1BP3 |
| EIF4G3 |
| ECE1 |
| USP48 |
| HSPG2 |
| E2F2 |
| MAN1C1 |
| SELENON |
| SH3BGRL3 |

| | |
|---|---|
| ZRANB2 | UBXN11 |
| ACADM | SLC9A1 |
| USP33 | MAP3K6 |
| DNAJB4 | GPR3 |
| IFI44L | AHDC1 |
| IFI44 | SERINC2 |
| CTBS | TMEM39B |
| MCOLN2 | KPNA6 |
| MCOLN3 | SFPQ |
| C1orf52 | CLSPN |
| SH3GLB1 | MAP7D1 |
| HS2ST1 | STK40 |
| GTF2B | CDCA8 |
| KYAT3 | INPP5B |
| GBP3 | FHL3 |
| GBP1 | UTP11 |
| GBP2 | BMP8A |
| GBP4 | BMP8B |
| GBP5 | LOC105378668 |
| GBP1P1 | NFYC |
| KIAA1107 | KCNQ4 |
| GLMN | CITED4 |
| BCAR3 | CTPS1 |
| FRRS1 | SCMH1 |
| STXBP3 | P3H1 |
| WDR47 | TIE1 |
| TMEM167B | CDC20 |
| GNAI3 | PTPRF |
| TMIGD3 | SLC6A9 |
| RAP1A | TMEM53 |
| ST7L | KIF2C |
| SLC22A15 | HPDL |
| ATP1A1-AS1 | NASP |
| SEC22B | RAD54L |
| LOC100996720 | STIL |
| FCGR1B | FOXD2-AS1 |
| SRGAP2-AS1 | ZYG11A |
| FCGR1CP | LRP8 |
| POLR3GL | CYB5RL |
| FCGR1A | SSBP3 |
| HIST2H4A | DEPDC1 |
| HIST2H2AA3 | HHLA3 |
| HIST2H2AA4 | DDAH1 |
| HIST2H4B | CDC7 |
| PLEKHO1 | F3 |

| | |
|---|---|
| APH1A | SASS6 |
| C1orf54 | GPR88 |
| HORMAD1 | CELSR2 |
| CTSS | PSRC1 |
| CTSK | GSTM4 |
| PSMD4 | GSTM2 |
| SNAPIN | CSF1 |
| RPS27 | TAFA3 |
| IL6R-AS1 | LINC01356 |
| IL6R | SLC16A1 |
| THBS3 | PTPN22 |
| RIT1 | DCLRE1B |
| SSR2 | IGSF3 |
| GLMP | PTGFRN |
| AIM2 | CD101 |
| CD48 | WARS2-AS1 |
| SLAMF7 | LINC00622 |
| CD244 | FAM72B |
| USF1 | FAM72C |
| TOMM40L | FAM72D |
| CREG1 | PIAS3 |
| ADCY10 | RBM8A |
| BLZF1 | LIX1L |
| RABGAP1L | ANKRD34A |
| COP1 | GJA5 |
| RALGPS2 | SF3B4 |
| SOAT1 | ANP32E |
| GLUL | CA14 |
| NPL | S100A16 |
| NCF2 | INTS3 |
| TRMT1L | KCNN3 |
| SWT1 | FAM189B |
| RGS1 | CLK2 |
| RGS13 | MEX3A |
| RGS2 | IQGAP3 |
| CFHR1 | GPATCH4 |
| NEK7 | NES |
| NR5A2 | PRCC |
| LINC00862 | KIRREL1 |
| CYB5R1 | IGSF8 |
| NENF | DCAF8 |
| ATF3 | SLAMF1 |
| DUSP10 | ADAMTS4 |
| AIDA | FCRLA |
| NVL | FCRLB |

| | |
|---|---|
| CNIH4 | C1orf226 |
| H3F3A | POGK |
| PSEN2 | RCSD1 |
| GUK1 | MPZL1 |
| AGT | SFT2D2 |
| ERO1B | FAM20B |
| SDCCAG8 | CACNA1E |
| ZNF124 | LAMC1 |
| CMPK2 | NMNAT2 |
| RSAD2 | PTGS2 |
| ID2 | ASPM |
| ADAM17 | KIF14 |
| TAF1B | KIF21B |
| PQLC3 | IGFN1 |
| YPEL5 | NAV1 |
| EIF2AK2 | IPO9 |
| HNRNPLL | MYBPH |
| MORN2 | ATP2B4 |
| SLC8A1 | TMCC2 |
| PRKCE | NUAK2 |
| PPP1R21 | SLC45A3 |
| PNPT1 | SLC41A1 |
| VRK2 | SLC26A9 |
| B3GNT2 | IL24 |
| UGP2 | FCMR |
| PELI1 | CD55 |
| AFTPH | CAMK1G |
| RAB1A | LAMB3 |
| PLEK | DTL |
| ARHGAP25 | PTPN14 |
| ANXA4 | CENPF |
| TIA1 | RRP15 |
| NAGK | PYCR2 |
| CAPG | PARP1 |
| VAMP8 | COQ8A |
| TMEM150A | CDC42BPA |
| ST3GAL5 | TARBP1 |
| KDM3A | IRF2BP2 |
| CYTOR | GPR137B |
| NEURL3 | MTR |
| LMAN2L | EXO1 |
| TSGA10 | TRIM58 |
| LIMS1 | TRAPPC12 |
| RALB | RNASEH1 |
| CXCR4 | ASAP2 |

| |
|---|
| ARHGAP15 |
| ORC4 |
| MMADHC |
| RND3 |
| NMI |
| TNFAIP6 |
| STAM2 |
| WDSUB1 |
| 07-Mar |
| TANK |
| IFIH1 |
| GCA |
| DHRS9 |
| SP3 |
| LINC01960 |
| CIR1 |
| NFE2L2 |
| RBM45 |
| PLEKHA3 |
| ITPRID2 |
| STAT1 |
| STAT4 |
| COQ10B |
| TYW5 |
| MAIP1 |
| SPATS2L |
| CLK1 |
| CYP20A1 |
| CCNYL1 |
| RPE |
| KANSL1L |
| TMBIM1 |
| DNAJB2 |
| DNPEP |
| TMEM198 |
| WDFY1 |
| MRPL44 |
| SP100 |
| HTR2B |
| ARMC9 |
| NPPC |
| ECEL1 |
| TRPM8 |
| ARL8B |
| EMC3 |

| |
|---|
| RRM2 |
| GREB1 |
| TRIB2 |
| EFR3B |
| DNMT3A |
| KIF3C |
| CENPA |
| TCF23 |
| CAD |
| GTF3C2 |
| IFT172 |
| WDR43 |
| GPATCH11 |
| DHX57 |
| OXER1 |
| PREPL |
| SPTBN1 |
| BCL11A |
| PCBP1 |
| ADD2 |
| CYP26B1 |
| RAB11FIP5 |
| TGOLN2 |
| C2orf68 |
| POLR1A |
| PROM2 |
| SNRNP200 |
| NCAPH |
| CNNM4 |
| MRPL30 |
| EIF5B |
| NPAS2 |
| IL1R2 |
| SH3RF3 |
| ANAPC1 |
| SLC20A1 |
| CKAP2L |
| IL1B |
| MARCO |
| STEAP3 |
| TMEM177 |
| TMEM185B |
| IWS1 |
| MYO7B |
| SMPD4 |

| |
|---|
| RAB5A |
| UBE2E1 |
| NKIRAS1 |
| NR1D2 |
| NGLY1 |
| LRRC3B |
| AZI2 |
| DYNC1LI1 |
| TRANK1 |
| ACAA1 |
| WDR48 |
| GORASP1 |
| CSRNP1 |
| CTNNB1 |
| ULK4 |
| SNRK |
| CCR1 |
| CCRL2 |
| LTF |
| IP6K2 |
| KLHDC8B |
| USP4 |
| TCTA |
| CYB561D2 |
| ACTR8 |
| SELENOK |
| HESX1 |
| KBTBD8 |
| TMF1 |
| CHMP2B |
| ZNF654 |
| C3orf38 |
| ST3GAL6 |
| TBC1D23 |
| SENP7 |
| CD47 |
| B4GALT4 |
| POGLUT1 |
| CD80 |
| ADPRH |
| HGD |
| IQCB1 |
| EAF2 |
| WDR5B |
| KPNA1 |

| |
|---|
| PTPN18 |
| FAM168B |
| MGAT5 |
| R3HDM1 |
| PKP4 |
| SPC25 |
| GORASP2 |
| DLX1 |
| DLX2 |
| MAP3K20 |
| CDCA7 |
| SCRN3 |
| MARS2 |
| KLF7 |
| FN1 |
| LINC00607 |
| LINC01963 |
| IGFBP5 |
| TTLL4 |
| ATG9A |
| CCL20 |
| PID1 |
| DNER |
| NCL |
| DGKD |
| HJURP |
| AGAP1 |
| ACKR3 |
| COL6A3 |
| MLPH |
| TWIST2 |
| GPC1 |
| FANCD2 |
| SYN2 |
| IQSEC1 |
| NUP210 |
| XPC |
| SLC6A6 |
| TGFBR2 |
| CMTM7 |
| ACVR2B |
| TRAK1 |
| VIPR1 |
| KIAA1143 |
| KIF15 |

| |
|---|
| PARP9 |
| PARP14 |
| ITGB5 |
| ZNF148 |
| TPRA1 |
| ABTB1 |
| SEC61A1 |
| ATP2C1 |
| NCK1 |
| ARMC8 |
| MRAS |
| PLSCR1 |
| CPB1 |
| GYG1 |
| HPS3 |
| RNF13 |
| TIPARP |
| PTX3 |
| RSRC1 |
| MFSD1 |
| SERPINI1 |
| GPR160 |
| TNFSF10 |
| DCUN1D1 |
| B3GNT5 |
| KLHL24 |
| MAP3K13 |
| DNAJB11 |
| RTP4 |
| TPRG1 |
| MB21D2 |
| FYTTD1 |
| SLC49A3 |
| C4orf48 |
| TNIP2 |
| MRFAP1 |
| SLC2A9 |
| HS3ST1 |
| BST1 |
| LAP3 |
| LCORL |
| CCDC149 |
| OCIAD2 |
| STAP1 |
| NPFFR2 |

| |
|---|
| TMEM158 |
| ALS2CL |
| NBEAL2 |
| PTPN23 |
| SMARCC1 |
| MAP4 |
| CDC25A |
| UCN2 |
| COL7A1 |
| NCKIPSD |
| PRKAR2A |
| NAA80 |
| CISH |
| ABHD14B |
| DUSP7 |
| NT5DC2 |
| FLNB |
| RPP14 |
| LRIG1 |
| FOXP1 |
| FAM86DP |
| DCBLD2 |
| FILIP1L |
| LINC01215 |
| BOC |
| QTRT2 |
| POLQ |
| CD86 |
| LINC02035 |
| MYLK |
| HEG1 |
| SLC12A8 |
| PLXNA1 |
| H1FX |
| NUDT16 |
| ACPP |
| TOPBP1 |
| WWTR1 |
| SMC4 |
| TRIM59 |
| TBL1XR1 |
| ZMAT3 |
| USP13 |
| DVL3 |
| ETV5 |

| |
|---|
| CXCL10 |
| CXCL11 |
| SCARB2 |
| STBD1 |
| CXCL13 |
| RASGEF1B |
| HELQ |
| MRPS18C |
| GPAT3 |
| HSD17B11 |
| SPP1 |
| PPM1K |
| HERC6 |
| HERC5 |
| TSPAN5 |
| EIF4E |
| METAP1 |
| ADH5 |
| DAPP1 |
| LAMTOR3 |
| DNAJB14 |
| LOC256880 |
| DDIT4L |
| UBE2D3-AS1 |
| INTS12 |
| MCUB |
| CASP6 |
| EGF |
| ALPK1 |
| CAMK2D |
| METTL14 |
| C4orf3 |
| MFSD8 |
| ABHD18 |
| C4orf33 |
| ELF2 |
| NDUFC1 |
| ZNF330 |
| IL15 |
| INPP4B |
| USP38 |
| PRMT9 |
| ARHGAP10 |
| GUCY1B1 |
| TDO2 |

| |
|---|
| XXYLT1 |
| DLG1 |
| FAM157A |
| IDUA |
| FGFRL1 |
| TACC3 |
| LETM1 |
| NSD2 |
| NELFA |
| NAT8L |
| NOP14 |
| LYAR |
| EVC |
| LOC93622 |
| TBC1D14 |
| AFAP1 |
| HTRA3 |
| ADGRA3 |
| STIM2 |
| GUF1 |
| SLC10A4 |
| DANCR |
| ERVMER34-1 |
| PDGFRA |
| COX18 |
| CXCL1 |
| CXCL5 |
| CXCL3 |
| CXCL2 |
| AREG |
| SDAD1 |
| SEPTIN11 |
| CCNI |
| SMARCAD1 |
| CENPE |
| SGMS2 |
| LEF1 |
| GAR1 |
| FAM241A |
| ANK2 |
| CCNA2 |
| PLK4 |
| SETD7 |
| MAML3 |
| ZNF827 |

| |
|---|
| CTSO |
| GLRB |
| GASK1B |
| TMEM144 |
| RXFP1 |
| FNIP2 |
| DDX60 |
| FBXO8 |
| AGA |
| CDKN2AIP |
| IRF2 |
| CASP3 |
| ACSL1 |
| MED10 |
| WDR70 |
| PTGER4 |
| ANXA2R |
| ARL15 |
| MAP3K1 |
| GPBP1 |
| TRIM23 |
| TRAPPC13 |
| SGTB |
| ERBIN |
| HEXB |
| GCNT4 |
| POLK |
| SCAMP1-AS1 |
| LHFPL2 |
| LYSMD3 |
| ARRDC3 |
| ARRDC3-AS1 |
| MCTP1 |
| ERAP1 |
| FAM174A |
| PAM |
| GIN1 |
| PJA2 |
| SLC25A46 |
| FEM1C |
| TMED7 |
| ATG12 |
| TNFAIP8 |
| PPIC |
| P4HA2 |

| |
|---|
| DCLK2 |
| SH3D19 |
| C4orf46 |
| NAF1 |
| LOC100506688 |
| NKD2 |
| ANKH |
| SKP2 |
| MCIDAS |
| DEPDC1B |
| CCNB1 |
| MCCC2 |
| MAP1B |
| UTP15 |
| ZBED3 |
| MTX3 |
| DHFR |
| MBLAC2 |
| POLR3G |
| FAM172A |
| RGMB |
| RGMB-AS1 |
| EFNA5 |
| STARD4-AS1 |
| LMNB1 |
| SLC12A2 |
| FBN2 |
| SHROOM1 |
| TCF7 |
| DDX46 |
| SPOCK1 |
| HNRNPA0 |
| KIF20A |
| TMEM173 |
| NRG2 |
| HBEGF |
| CD14 |
| PCDHB5 |
| PCDHB6 |
| PCDHGA12 |
| DIAPH1 |
| ARAP3 |
| SPRY4 |
| TCERG1 |
| GRPEL2 |

| |
|---|
| SLC22A4 |
| IRF1 |
| ZCCHC10 |
| UBE2B |
| TGFBI |
| CTNNA1 |
| PAIP2 |
| CXXC5-AS1 |
| CYSTM1 |
| PCDHAC2 |
| TAF7 |
| GNPDA1 |
| NR3C1 |
| CSNK1A1 |
| DCTN4 |
| SMIM3 |
| ATOX1 |
| HAVCR2 |
| MAT2B |
| UBTD2 |
| CREBRF |
| TMED9 |
| PHYKPL |
| ZNF879 |
| SQSTM1 |
| RNF130 |
| MGAT1 |
| HEIH |
| LINC00847 |
| SERPINB1 |
| PXDC1 |
| NRN1 |
| LY86 |
| SLC35B3 |
| CD83 |
| DTNBP1 |
| GMPR |
| FAM8A1 |
| TDP2 |
| HIST1H2BC |
| HIST1H2AC |
| HIST1H2BD |
| BTN3A1 |
| BTN3A3 |
| BTN2A1 |

| |
|---|
| PCYOX1L |
| PDGFRB |
| CAMK2A |
| TCOF1 |
| SYNPO |
| GPX3 |
| TNIP1 |
| LARP1 |
| GEMIN5 |
| ADAM19 |
| RNF145 |
| PTTG1 |
| HRH2 |
| SIMC1 |
| RNF44 |
| UNC5A |
| FGFR4 |
| GRK6 |
| PRR7 |
| DBN1 |
| PDLIM7 |
| DOK3 |
| HNRNPAB |
| COL23A1 |
| HNRNPH1 |
| MGAT4B |
| GFPT2 |
| BTNL9 |
| TRIM41 |
| LYRM4-AS1 |
| TFAP2A |
| PHACTR1 |
| GFOD1 |
| ATXN1 |
| NRSN1 |
| DCDC2 |
| PRR3 |
| ATAT1 |
| C6orf136 |
| NRM |
| MDC1 |
| TCF19 |
| LTA |
| TNF |
| BAG6 |

| |
|---|
| ZNF322 |
| HLA-A |
| HLA-L |
| HLA-E |
| HLA-B |
| HCP5 |
| DDAH2 |
| CLIC1 |
| HSPA1A |
| HSPA1B |
| SNHG32 |
| NEU1 |
| STK19 |
| HLA-DRB5 |
| HLA-DRB1 |
| HLA-DQB1 |
| PSMB8 |
| PSMB9 |
| TAP1 |
| HLA-DPA1 |
| B3GALT4 |
| BAK1 |
| ILRUN |
| ETV7 |
| RNF8 |
| CMTR1 |
| TREM2 |
| MAD2L1BP |
| NFKBIE |
| ENPP4 |
| GCLC |
| ADGRB3 |
| LMBRD1 |
| OGFRL1 |
| CGAS |
| SLC17A5 |
| LOC101928489 |
| CD109 |
| HMGN3-AS1 |
| CYB5R4 |
| MRAP2 |
| SNX14 |
| SLC35A1 |
| CNR1 |
| RRAGD |

| |
|---|
| EHMT2 |
| ZBTB12 |
| ATF6B |
| PRRT1 |
| AGPAT1 |
| KIFC1 |
| HMGA1 |
| PPARD |
| FANCE |
| KCNK5 |
| FOXP4 |
| FRS3 |
| TRERF1 |
| RPL7L1 |
| CUL7 |
| XPO5 |
| POLH |
| VEGFA |
| LOC101929705 |
| C6orf223 |
| TMEM63B |
| SLC29A1 |
| MCM3 |
| TRAM2 |
| DST |
| KCNQ5 |
| COL12A1 |
| SYNCRIP |
| PM20D2 |
| MDN1 |
| CRYBG1 |
| FYN |
| HBS1L |
| MTFR2 |
| BCLAF1 |
| NHSL1 |
| REPS1 |
| TXLNB |
| CITED2 |
| ADAT2 |
| LTV1 |
| SASH1 |
| NUP43 |
| ULBP3 |
| SYNE1 |

| |
|---|
| ATG5 |
| OSTM1 |
| SESN1 |
| CD164 |
| SLC22A16 |
| SLC16A10 |
| MFSD4B |
| NCOA7 |
| RNF146 |
| STX7 |
| HEBP2 |
| ABRACL |
| FILNC1 |
| FUCA2 |
| STX11 |
| GINM1 |
| TMEM242 |
| DYNLT1 |
| TAGAP |
| WTAP |
| RNASET2 |
| ZFAND2A |
| C1GALT1 |
| HDAC9 |
| GPNMB |
| SNX10 |
| ZNRF2 |
| NT5C3A |
| RALA |
| STK17A |
| BLVRA |
| HUS1 |
| ZNF273 |
| CCT6P3 |
| ZNF92 |
| GS1-124K5.11 |
| STAG3L4 |
| GTF2IRD2B |
| POR |
| PTPN12 |
| TMEM60 |
| CD36 |
| ABCB1 |
| GTPBP10 |
| GATAD1 |

| |
|---|
| SYNJ2 |
| AFDN |
| KIF25-AS1 |
| DNAAF5 |
| SUN1 |
| INTS1 |
| ELFN1-AS1 |
| EIF3B |
| LFNG |
| RADIL |
| ACTB |
| FSCN1 |
| MACC1 |
| IL6 |
| HNRNPA2B1 |
| FKBP9 |
| ANLN |
| ELMO1 |
| TRG-AS1 |
| POLM |
| CAMK2B |
| OGDH |
| IGFBP3 |
| LOC730338 |
| FKBP9P1 |
| POM121 |
| MLXIPL |
| POM121C |
| HIP1 |
| CCL24 |
| YWHAG |
| DTX2 |
| SEMA3A |
| TAF6 |
| SPDYE3 |
| EPHB4 |
| SRRT |
| ACHE |
| SERPINE1 |
| PLOD3 |
| CUX1 |
| RASA4 |
| PUS7 |
| LAMB1 |
| RBM28 |

| | |
|---|---|
| RBM48 | IMPDH1 |
| SAMD9 | CALU |
| SAMD9L | TMEM209 |
| BRI3 | LUC7L2 |
| ZNF394 | HIPK2 |
| ZNF655 | TBXAS1 |
| ZNF3 | KIAA1147 |
| GNB2 | MGAM |
| TRIP6 | CLEC5A |
| PRKRIP1 | CASP2 |
| PSMC2 | ZYX |
| SYPL1 | ASIC3 |
| HBP1 | XRCC2 |
| BCAP29 | ACTR3B |
| DNAJB9 | PAXIP1 |
| ZNF277 | NOM1 |
| IFRD1 | UBE3C |
| BMT2 | PLCXD1 |
| TFEC | PRKX |
| CAPZA2 | STS |
| POT1 | TBL1X |
| GCC1 | WWC3 |
| STRIP2 | CA5BP1 |
| LINC-PINT | GRPR |
| MKLN1-AS | TXLNG |
| PARP12 | SH3KBP1 |
| MKRN1 | POLA1 |
| ADCK2 | CYBB |
| PDIA4 | MED14 |
| GIMAP2 | RBM10 |
| SMARCD3 | UBA1 |
| NUB1 | CDK16 |
| DNAJB6 | SYN1 |
| DHRSX | TIMP1 |
| TMSB4X | SUV39H1 |
| RAB9A | PIM2 |
| SAT1 | PLP2 |
| CHST7 | PRICKLE3 |
| RP2 | TSPYL2 |
| JADE3 | SMC1A |
| LOC107985688 | HUWE1 |
| ITGB1BP2 | FAM120C |
| UPRT | FGD1 |
| GLA | GNL3L |
| ARMCX1 | APEX2 |

| |
|---|
| ARMCX3 |
| RBM41 |
| PRPS1 |
| NXT2 |
| ACSL4 |
| LONRF3 |
| UBE2A |
| ZBTB33 |
| LAMP2 |
| C1GALT1C1 |
| STAG2 |
| RAP2C |
| INTS6L |
| RENBP |
| TKTL1 |
| ATP6AP1 |
| MPP1 |
| MSR1 |
| PDGFRL |
| ASAH1 |
| BIN3 |
| PEBP4 |
| ADAM28 |
| PPP2R2A |
| PNMA2 |
| PNOC |
| GTF2E2 |
| BRF2 |
| PLEKHA2 |
| TM2D2 |
| FNTA |
| CEBPD |
| ATP6V1H |
| RGS20 |
| LYN |
| RPS20 |
| FAM110B |
| SDCBP |
| VCPIP1 |
| SGK3 |
| COPS5 |
| GDAP1 |
| HNF4G |
| FABP5 |
| SNX16 |

| |
|---|
| ZC3H12B |
| AR |
| EFNB1 |
| KIF4A |
| NONO |
| SLC16A2 |
| RTL3 |
| APOOL |
| PCDH19 |
| COL4A5 |
| AMMECR1 |
| RTL9 |
| UTP14A |
| BCORL1 |
| MBNL3 |
| RTL8B |
| RTL8C |
| RTL8A |
| MMGT1 |
| MAP7D3 |
| ARHGEF6 |
| MAMLD1 |
| L1CAM |
| G6PD |
| DKC1 |
| F8A1 |
| F8A2 |
| F8A3 |
| KBTBD11 |
| PRAG1 |
| CSGALNACT1 |
| LZTS1 |
| REEP4 |
| BMP1 |
| SLC39A14 |
| TNFRSF10D |
| LOXL2 |
| STC1 |
| DPYSL2 |
| PBK |
| TEX15 |
| NRG1 |
| MAK16 |
| ADGRA2 |
| RAB11FIP1 |

| |
|---|
| ATP6V0D2 |
| LOC101929709 |
| NBN |
| DECR1 |
| PLEKHF2 |
| PTDSS1 |
| SDC2 |
| CPQ |
| RNF19A |
| ATP6V1C1 |
| DPYS |
| LRP12 |
| OXR1 |
| ENY2 |
| ZHX1 |
| FBXO32 |
| NSMCE2 |
| FAM49B |
| LY6E |
| GSDMD |
| COMMD5 |
| CDC37L1 |
| JAK2 |
| CD274 |
| PDCD1LG2 |
| TTC39B |
| PLIN2 |
| DENND4C |
| IFNB1 |
| PLAA |
| IFT74 |
| DDX58 |
| APTX |
| DNAJA1 |
| BAG1 |
| CHMP5 |
| UBAP1 |
| CCDC107 |
| CREB3 |
| RECK |
| GLIPR2 |
| ZCCHC7 |
| C9orf85 |
| LINC01504 |
| IDNK |

| |
|---|
| FGFR1 |
| GINS4 |
| HOOK3 |
| PRKDC |
| IMPAD1 |
| RRS1 |
| MYBL1 |
| MSC |
| PABPC1 |
| DERL1 |
| ATAD2 |
| MTSS1 |
| TRIB1 |
| MYC |
| KCNQ3 |
| CHRAC1 |
| DENND3 |
| ARC |
| GLI4 |
| TIGD5 |
| PYCR3 |
| FAM83H |
| SCRIB |
| PLEC |
| GPAA1 |
| HGH1 |
| HSF1 |
| CPSF1 |
| SLC39A4 |
| TONSL |
| PPP1R16A |
| RECQL4 |
| ARHGAP39 |
| SMARCA2 |
| VLDLR |
| GLIS3 |
| IL33 |
| GLDC |
| PSIP1 |
| NOL6 |
| UBAP2 |
| RUSC2 |
| ALDH1B1 |
| FAM27E3 |
| APBA1 |

| |
|---|
| KIF27 |
| CTSL |
| CDK20 |
| NFIL3 |
| ZNF484 |
| CARD19 |
| FAM120AOS |
| SLC35D2 |
| PRXL2C |
| TDRD7 |
| TBC1D2 |
| NR4A3 |
| ERP44 |
| ZNF189 |
| SLC31A2 |
| CDC26 |
| ATP6V1G1 |
| NEK6 |
| HSPA5 |
| ZNF79 |
| SLC25A25 |
| PTPA |
| TOR1B |
| TOR1A |
| UCK1 |
| AGPAT2 |
| SNHG7 |
| TRAF2 |
| FBXW5 |
| IFITM1 |
| IFITM3 |
| IRF7 |
| MOB2 |
| CDKN1C |
| TRIM21 |
| TRIM5 |
| TRIM22 |
| TMEM9B |
| ZNF143 |
| ADM |
| GTF2H1 |
| ANO5 |
| CD59 |
| LMO2 |
| EXT2 |

| |
|---|
| CARNMT1 |
| GNAQ |
| SHC3 |
| SYK |
| BICD2 |
| FAM120A |
| FBP1 |
| PTCH1 |
| ZNF367 |
| MFSD14C |
| SMC2 |
| PTPN3 |
| ECPAS |
| TNFSF15 |
| TNC |
| PHF19 |
| CRB2 |
| LHX2 |
| STXBP1 |
| SH2D3C |
| CIZ1 |
| DNM1 |
| GOLGA2 |
| URM1 |
| CERCAM |
| ODF2 |
| NUP188 |
| NCS1 |
| FIBCD1 |
| GTF3C5 |
| SARDH |
| BRD3 |
| WDR5 |
| FCN1 |
| MRPS2 |
| NACC2 |
| QSOX2 |
| GPSM1 |
| CARD9 |
| RABL6 |
| ABCA2 |
| SAPCD2 |
| TPRN |
| RNF208 |
| TUBB4B |

| |
|---|
| CRY2 |
| PEX16 |
| HARBI1 |
| ZNF408 |
| NR1H3 |
| UBE2L6 |
| STX3 |
| CYB561A3 |
| POLR2G |
| PLAAT4 |
| BATF2 |
| KAT5 |
| EFEMP2 |
| DRAP1 |
| BRMS1 |
| UNC93B1 |
| CHKA |
| RNF121 |
| FOLR2 |
| SPCS2 |
| UVRAG |
| CTSC |
| ARHGAP42 |
| BIRC2 |
| TMEM123 |
| CASP4 |
| CASP5 |
| CASP1 |
| CARD16 |
| MSANTD4 |
| RDX |
| ZC3H12C |
| FDX1 |
| PPP2R1B |
| RBM7 |
| CADM1 |
| TBCEL |
| TBRG1 |
| KCNJ1 |
| KLF6 |
| KIN |
| USP6NL |
| OPTN |
| HSPA14 |
| ARL5B |

| |
|---|
| NRARP |
| NSMF |
| BET1L |
| TMEM80 |
| PIDD1 |
| PNPLA2 |
| MUC5AC |
| H19 |
| PHLDA2 |
| STIM1 |
| TIMM10B |
| ILK |
| TUB |
| DENND5A |
| MICAL2 |
| NCR3LG1 |
| E2F8 |
| NAV2 |
| LINC00294 |
| CD44 |
| FJX1 |
| TRIM44 |
| ALX4 |
| TSPAN18 |
| TP53I11 |
| CREB3L1 |
| ARHGAP1 |
| CKAP5 |
| DDB2 |
| TNKS1BP1 |
| FAM111B |
| CCDC86 |
| CPSF7 |
| MYRF |
| FEN1 |
| FADS2 |
| INCENP |
| EML3 |
| HNRNPUL2 |
| C11orf95 |
| FLRT1 |
| GPR137 |
| MEN1 |
| EHD1 |
| SNX32 |

| |
|---|
| DNAJC1 |
| BMI1 |
| MSRB2 |
| YME1L1 |
| ZNF438 |
| ZEB1 |
| CUL2 |
| CREM |
| CSGALNACT2 |
| LNCAROD |
| UBE2D1 |
| ZNF365 |
| NRBF2 |
| SIRT1 |
| SRGN |
| VPS26A |
| FAM241B |
| AIFM2 |
| PSAP |
| MICU1 |
| PTEN |
| LIPA |
| IFIT2 |
| IFIT3 |
| IFIT1 |
| IFIT5 |
| PCGF5 |
| HECTD2 |
| TNKS2 |
| 05-Mar |
| PIK3AP1 |
| GOLGA7B |
| SLC25A28 |
| BLOC1S2 |
| PKD2L1 |
| KCNIP2 |
| BORCS7 |
| NT5C2 |
| SFR1 |
| GSTO1 |
| DUSP5 |
| SHOC2 |
| ACSL5 |
| CASP7 |
| RGS10 |

| |
|---|
| EIF1AD |
| PACS1 |
| KLC2 |
| RAB1B |
| SLC29A2 |
| PELI3 |
| RBM14 |
| SPTBN2 |
| PC |
| GRK2 |
| CLCF1 |
| CDK2AP2 |
| FAM86C2P |
| C11orf24 |
| LRP5 |
| ALG1L9P |
| FAM86C1 |
| LOC100287837 |
| ARHGEF17 |
| UCP2 |
| ARRB1 |
| DGAT2 |
| GVQW3 |
| DDIAS |
| NAALAD2 |
| AMOTL1 |
| DIXDC1 |
| PAFAH1B2 |
| RNF214 |
| BACE1 |
| H2AFX |
| C2CD2L |
| CBL |
| RNF26 |
| NECTIN1 |
| OAF |
| MIR100HG |
| SPA17 |
| NRGN |
| VSIG2 |
| STT3A |
| DCPS |
| ADAMTS8 |
| LOC103611081 |
| NCAPD3 |

| |
|---|
| PPP2R2D |
| NINJ2 |
| PARP11 |
| CD27-AS1 |
| TAPBPL |
| ING4 |
| GPR162 |
| C1S |
| PEX5 |
| C3AR1 |
| M6PR |
| CLEC2B |
| GABARAPL1 |
| CDKN1B |
| GPRC5A |
| HEBP1 |
| GSG1 |
| EMP1 |
| MED21 |
| CAPRIN2 |
| H3F3C |
| YAF2 |
| IRAK4 |
| TWF1 |
| LOC105369748 |
| RND1 |
| LMBR1L |
| CERS5 |
| SLC11A2 |
| LETMD1 |
| CSRNP2 |
| IGFBP6 |
| ITGB7 |
| PRR13 |
| SMUG1 |
| BLOC1S1 |
| CD63 |
| LOC105369779 |
| MYL6 |
| NAB2 |
| STAC3 |
| DDIT3 |
| OS9 |
| TSPAN31 |
| USP15 |

| |
|---|
| ZMYND11 |
| GTPBP4 |
| NET1 |
| PFKFB3 |
| CAMK1D |
| MCM10 |
| FAM171A1 |
| VIM |
| PDSS1 |
| SVIL |
| JCAD |
| CCNY |
| BMS1 |
| ALOX5 |
| WDFY4 |
| VSTM4 |
| ZWINT |
| HNRNPH3 |
| DDX21 |
| COL13A1 |
| EIF4EBP2 |
| PALD1 |
| UNC5B |
| SLC29A3 |
| VSIR |
| SEC24C |
| VCL |
| ZCCHC24 |
| TSPAN14 |
| CDHR1 |
| KIF20B |
| KIF11 |
| HHEX |
| MYOF |
| CEP55 |
| HELLS |
| FRAT2 |
| RRP12 |
| PYROXD2 |
| SCD |
| OLMALINC |
| TWNK |
| PDZD7 |
| SFXN3 |
| TLX1 |

| |
|---|
| MON2 |
| LYZ |
| TBC1D15 |
| NAP1L1 |
| BBS10 |
| OSBPL8 |
| CSRP2 |
| KITLG |
| POC1B-GALNT4 |
| LINC01619 |
| BTG1 |
| VEZT |
| SCYL2 |
| WASHC4 |
| CORO1C |
| USP30 |
| C12orf76 |
| ARPC3 |
| ERP29 |
| TRAFD1 |
| OAS1 |
| OAS2 |
| SDS |
| SDSL |
| MAP1LC3B2 |
| PRKAB1 |
| ACADS |
| OASL |
| P2RX7 |
| P2RX4 |
| WDR66 |
| GTF2H3 |
| UBC |
| ZNF26 |
| ZMYM5 |
| MICU2 |
| POMP |
| USPL1 |
| STARD13 |
| ALG5 |
| SUPT20H |
| ELF1 |
| WBP4 |
| MTRF1 |
| EPSTI1 |

| |
|---|
| PPRC1 |
| NOLC1 |
| NFKB2 |
| PDCD11 |
| CALHM2 |
| SH3PXD2A |
| SMC3 |
| PDCD4 |
| LOC103344931 |
| TCF7L2 |
| HSPA12A |
| NANOS1 |
| EIF3A |
| TACC2 |
| MKI67 |
| EBF3 |
| ADAM8 |
| TUBGCP2 |
| SLC6A12 |
| LINC00942 |
| FBXL14 |
| FOXM1 |
| TULP3 |
| TIGAR |
| VWF |
| NCAPD2 |
| MLF2 |
| PTMS |
| CD4 |
| CDCA3 |
| ATN1 |
| YBX3 |
| APOLD1 |
| WBP11 |
| BHLHE41 |
| SSPN |
| DDX11 |
| BICD1 |
| SLC38A1 |
| SLC48A1 |
| VDR |
| ADCY6 |
| TUBA1C |
| TROAP |
| KCNH3 |

| |
|---|
| SLC25A30 |
| COG3 |
| ITM2B |
| RB1 |
| LPAR6 |
| RCBTB2 |
| LINC00462 |
| FNDC3A |
| SETDB2 |
| PHF11 |
| DLEU2 |
| TRIM13 |
| INTS6 |
| EDNRB |
| UBAC2-AS1 |
| UBAC2 |
| GPR18 |
| TNFSF13B |
| RAB20 |
| ANKRD10 |
| THTPA |
| PSME1 |
| PSME2 |
| TINF2 |
| RIPK3 |
| CTSG |
| SNX6 |
| SRP54 |
| PPP2R3C |
| SEC23A |
| TRAPPC6B |
| RN7SL1 |
| RN7SL3 |
| TMX1 |
| PSMC6 |
| DDHD1 |
| FBXO34 |
| AP5M1 |
| PSMA3 |
| SIX1 |
| SLC38A6 |
| LOC105370526 |
| ZBTB1 |
| CHURC1 |
| MPP5 |

| |
|---|
| ASIC1 |
| POU6F1 |
| SMAGP |
| ACVR1B |
| KRT7 |
| KRT79 |
| ESPL1 |
| SP1 |
| HOXC10 |
| CBX5 |
| GDF11 |
| RAB5B |
| IKZF4 |
| ANKRD52 |
| CS |
| TIMELESS |
| NXPH4 |
| R3HDM2 |
| MBD6 |
| CTDSP2 |
| SLC16A7 |
| CPSF6 |
| ZFC3H1 |
| ATP2B1 |
| ELK3 |
| SLC9A7P1 |
| TMPO-AS1 |
| TMPO |
| UTP20 |
| NT5DC3 |
| CHST11 |
| ALDH1L2 |
| POLR3B |
| CMKLR1 |
| TMEM119 |
| GLTP |
| TCHP |
| ATXN2 |
| PTPN11 |
| DDX54 |
| IQCD |
| TPCN1 |
| RNFT2 |
| FBXW8 |
| CIT |

| | |
|---|---|
| ATP6V1D | GCN1 |
| PIGH | PXN |
| RAD51B | CAMKK2 |
| DCAF5 | MLXIP |
| MED6 | CLIP1 |
| ZFYVE1 | VPS37B |
| ACOT1 | KMT5A |
| ACOT2 | NCOR2 |
| ACOT4 | BRI3BP |
| NPC2 | STX2 |
| IFT43 | SFSWAP |
| SNW1 | ULK1 |
| PSMC1 | EP400 |
| CALM1 | NOC4L |
| ATXN3 | FBRSL1 |
| IFI27 | POLE |
| PPP2R5C | PGAM5 |
| HSP90AA1 | SKA3 |
| WDR20 | SACS |
| EIF5 | CENPJ |
| PLD4 | SLC7A1 |
| AVEN | KATNAL1 |
| EMC7 | RFC3 |
| KATNBL1 | ZC3H13 |
| RMDN3 | FAM124A |
| TMEM87A | DIAPH3 |
| UBR1 | TBC1D4 |
| TMEM62 | LMO7 |
| PDIA3 | RBM26 |
| PATL2 | SLITRK5 |
| B2M | ABCC4 |
| TRIM69 | FARP1 |
| SQOR | STK24 |
| GALK2 | SOX1 |
| TNFAIP8L3 | SUPT16H |
| CYP19A1 | LTB4R |
| LYSMD2 | PAX9 |
| MAPK6 | PNN |
| FAM214A | SOS2 |
| PIGB | MAP4K5 |
| LOC145783 | NIN |
| TCF12 | DLGAP5 |
| ADAM10 | DACT1 |
| LDHAL6B | RTN1 |
| LACTB | SYNJ2BP |

| |
|---|
| RPS27L |
| RAB8B |
| CIAO2A |
| PPIB |
| TRIP4 |
| OAZ2 |
| FEM1B |
| PARP6 |
| HEXA |
| BBS4 |
| ADPGK |
| COMMD4 |
| TSPAN3 |
| PSMA4 |
| CTSH |
| BCL2A1 |
| ZFAND6 |
| RAMAC |
| NMB |
| SEC11A |
| KLHL25 |
| ISG20 |
| HAPLN3 |
| FES |
| LINS1 |
| SELENOS |
| TM2D3 |
| MRPL28 |
| TMEM8A |
| MCRIP2 |
| C16orf91 |
| MSRB1 |
| AMDHD2 |
| ZNF200 |
| TXNDC11 |
| NTAN1 |
| ARL6IP1 |
| GDE1 |
| ACSM5 |
| TMEM159 |
| SMG1P3 |
| SMG1P1 |
| OTOAP1 |
| NUPR1 |
| ASPHD1 |

| |
|---|
| DCAF4 |
| NUMB |
| PNMA1 |
| LTBP2 |
| YLPM1 |
| JDP2 |
| GPATCH2L |
| VASH1 |
| CEP128 |
| FLRT2 |
| RIN3 |
| SYNE3 |
| CCDC85C |
| TNFAIP2 |
| XRCC3 |
| JAG2 |
| MTA1 |
| HERC2 |
| ARHGAP11A |
| SLC12A6 |
| LPCAT4 |
| C15orf41 |
| BUB1B |
| PLCB2 |
| ANKRD63 |
| INAFM2 |
| CHST14 |
| KNL1 |
| CHAC1 |
| RPAP1 |
| MYEF2 |
| ARPP19 |
| MNS1 |
| AQP9 |
| TLN2 |
| ZNF609 |
| RBPMS2 |
| IGDCC4 |
| SMAD6 |
| SMAD3 |
| MAP2K5 |
| CORO2B |
| ANP32A |
| PAQR5 |
| KIF23 |

| | |
|---|---|
| TMEM219 | STRA6 |
| CD2BP2 | UBL7 |
| ZNF688 | C15orf39 |
| STX4 | SIN3A |
| ZNF267 | CSPG4 |
| ITFG1 | TBC1D2B |
| CYLD | CRABP1 |
| CES1 | ADAMTS7 |
| NDRG4 | IL16 |
| TRADD | ADAMTS7P1 |
| LRRC29 | ABHD2 |
| PSMB10 | POLG |
| COG4 | TICRR |
| IST1 | ANPEP |
| MAP1LC3B | AP3S2 |
| CYBA | BLM |
| RILP | SV2B |
| MIR22HG | SLCO3A1 |
| PAFAH1B1 | RGMA |
| USP6 | IGF1R |
| DERL2 | PCSK6 |
| XAF1 | TARSL2 |
| CHRNB1 | RHBDF1 |
| EIF4A1 | NPRL3 |
| ADPRM | CAPN15 |
| TVP23B | PIGQ |
| SNORD3A | JMJD8 |
| DHRS7B | RPUSD1 |
| SDF2 | MAPK8IP3 |
| CRLF3 | ZNF598 |
| TEFM | SLC9A3R2 |
| RNF135 | TSC2 |
| EVI2A | RNPS1 |
| UTP6 | ABCA3 |
| CCL23 | CCNF |
| CCL18 | TEDC2 |
| CCL3 | PKMYT1 |
| CCL4 | PAQR4 |
| ZNHIT3 | TFAP4 |
| CCR7 | GLIS2 |
| CNP | VASN |
| DHX58 | UBALD1 |
| BECN1 | MGRN1 |
| PTGES3L | C16orf72 |
| IFI35 | GSPT1 |

| |
|---|
| LINC02210 |
| GOSR2 |
| COPZ2 |
| SNX11 |
| CALCOCO2 |
| UBE2Z |
| MRPL27 |
| C17orf67 |
| PPM1E |
| SMG8 |
| TUBD1 |
| APPBP2 |
| TLK2 |
| PRR29 |
| ICAM2 |
| AMZ2P1 |
| GNA13 |
| ABCA5 |
| SLC39A11 |
| SSTR2 |
| FAM104A |
| GPRC5C |
| CD300A |
| SAP30BP |
| H3F3B |
| SPHK1 |
| ST6GALNAC2 |
| EIF4A3 |
| CHMP6 |
| YES1 |
| MYL12A |
| MYL12B |
| RAB12 |
| MPPE1 |
| ESCO1 |
| ABHD3 |
| RIOK3 |
| OSBPL1A |
| TRAPPC8 |
| RNF138 |
| ZNF271P |
| C18orf21 |
| PMAIP1 |
| PIGN |
| CNDP2 |

| |
|---|
| LINC02185 |
| MRTFB |
| MPV17L |
| XYLT1 |
| KNOP1 |
| EEF2K |
| COG7 |
| PLK1 |
| IL21R |
| GTF3C1 |
| ATXN2L |
| SPN |
| SEZ6L2 |
| KCTD13 |
| TBX6 |
| YPEL3 |
| SEPTIN1 |
| SETD1A |
| BCKDK |
| FUS |
| ITGAM |
| TGFB1I1 |
| SHCBP1 |
| ORC6 |
| FTO |
| IRX3 |
| OGFOD1 |
| MT2A |
| CPNE2 |
| CCL22 |
| ADGRG1 |
| CNOT1 |
| CDH5 |
| CMTM4 |
| ZDHHC1 |
| RIPOR1 |
| ACD |
| SLC7A6 |
| PDPR |
| MTSS2 |
| DHODH |
| FA2H |
| GSE1 |
| SLC7A5 |
| ZFPM1 |

| |
|---|
| MBP |
| HSBP1L1 |
| RBCK1 |
| PANK2 |
| CDS2 |
| ABHD12 |
| ID1 |
| PDRG1 |
| TRPC4AP |
| EDEM2 |
| PROCR |
| RAB5IF |
| CTNNBL1 |
| ADA |
| SYS1 |
| CD40 |
| RNF114 |
| RTF2 |
| ATP5F1E |
| ARFRP1 |
| AZU1 |
| PRTN3 |
| ABHD17A |
| GADD45B |
| MFSD12 |
| DAPK3 |
| VAV1 |
| MCOLN1 |
| 02-Mar |
| ZNF266 |
| ANGPTL6 |
| TMED1 |
| TIMM29 |
| ZNF627 |
| ACP5 |
| ZNF823 |
| ZNF700 |
| ZNF433 |
| ZNF136 |
| ZNF442 |
| JUNB |
| DNASE2 |
| CALR |
| HSH2D |
| FAM32A |

| |
|---|
| ZC3H18 |
| CDT1 |
| CBFA2T3 |
| ZNF778 |
| VPS9D1-AS1 |
| SPIRE2 |
| DBNDD1 |
| FAM57A |
| GEMIN4 |
| PRPF8 |
| DPH1 |
| MNT |
| HASPIN |
| P2RX1 |
| ATP2A3 |
| MYBBP1A |
| PELP1 |
| ARRB2 |
| MINK1 |
| CAMTA2 |
| DHX33 |
| NLRP1 |
| DLG4 |
| CTDNEP1 |
| NLGN2 |
| KDM6B |
| TMEM88 |
| CHD3 |
| CNTROB |
| ALOXE3 |
| VAMP2 |
| PFAS |
| PIK3R6 |
| NTN1 |
| ARHGAP44 |
| ELAC2 |
| PMP22 |
| MPRIP |
| SREBF1 |
| LLGL1 |
| SPECC1 |
| KSR1 |
| NOS2 |
| TMEM97 |
| SPAG5 |

| |
|---|
| SMIM7 |
| BST2 |
| GDF15 |
| BORCS8 |
| ZNF738 |
| ZNF675 |
| C19orf12 |
| HAMP |
| PSENEN |
| ZNF565 |
| GMFG |
| C19orf47 |
| SERTAD1 |
| CEACAM21 |
| RABAC1 |
| PINLYP |
| ZNF230 |
| ZNF222 |
| ZNF224 |
| ZNF227 |
| RELB |
| MEIS3 |
| NAPA |
| SELENOW |
| HSD17B14 |
| BAX |
| FTL |
| TRPM4 |
| FCGRT |
| NOSIP |
| IL4I1 |
| JOSD2 |
| NKG7 |
| ZNF613 |
| ZNF350 |
| ZNF766 |
| ZNF600 |
| ZNF468 |
| LILRA5 |
| LILRA2 |
| ZNF547 |
| ZNF211 |
| LINC00278 |
| ATP6V1E1 |
| BCL2L13 |

| |
|---|
| KIAA0100 |
| FLOT2 |
| PHF12 |
| ANKRD13B |
| CCL1 |
| TAF15 |
| MYO19 |
| ACACA |
| SOCS7 |
| SRCIN1 |
| STAC2 |
| IKZF3 |
| CSF3 |
| THRA |
| CASC3 |
| RARA |
| TOP2A |
| JUP |
| ATP6V0A1 |
| COASY |
| PLEKHH3 |
| CNTNAP1 |
| PSME3 |
| BRCA1 |
| ETV4 |
| LSM12 |
| C17orf53 |
| UBTF |
| KIF18B |
| C1QL1 |
| DCAKD |
| PLCD3 |
| MAP3K14-AS1 |
| MAPT |
| THCAT158 |
| SP2 |
| DLX4 |
| DLX3 |
| COL1A1 |
| XYLT2 |
| EME1 |
| ANKRD40 |
| MBTD1 |
| AKAP1 |
| SRSF1 |

| | |
|---|---|
| USP18 | TSPOAP1-AS1 |
| MRPL40 | PRR11 |
| DGCR6L | TANC2 |
| USP41 | MAP3K3 |
| SDF2L1 | PECAM1 |
| HSCB | PRKCA |
| EMID1 | SLC16A6 |
| RHBDD3 | CDC42EP4 |
| TCN2 | SLC9A3R1 |
| RTCB | CDR2L |
| HMOX1 | ITGB4 |
| NCF4 | WBP2 |
| APOBEC3G | TRIM47 |
| RPS19BP1 | TRIM65 |
| RBX1 | SRSF2 |
| NAGA | SEPTIN9 |
| POLDIP3 | SOCS3 |
| BIK | TIMP2 |
| PIM3 | C1QTNF1 |
| TYMP | CBX2 |
| ODF3B | CBX4 |
| CHKB-DT | TBC1D16 |
| ARSA | RPTOR |
| SIK1B | LOC100129503 |
| SAMSN1 | FASN |
| USP25 | FOXK2 |
| C21orf91 | FN3K |
| LINC00158 | TBCD |
| MIR155HG | COLEC12 |
| OLIG2 | EPB41L3 |
| IFNAR1 | SNRPD1 |
| IFNGR2 | TMEM241 |
| TMEM50B | ZNF521 |
| DONSON | CDH2 |
| SETD4 | RNF125 |
| CBR1 | MAPRE2 |
| VPS26C | SIGLEC15 |
| KCNJ6 | RNF165 |
| ETS2 | SKA1 |
| BRWD1 | C18orf54 |
| BACE2 | NEDD4L |
| MX2 | SERPINB2 |
| MX1 | SOX12 |
| SIK1 | NRSN2 |
| PDXK | CSNK2A1 |

| |
|---|
| **PTTG1IP** |

| |
|---|
| **FAM110A** |
| **PCED1A** |
| **ATRN** |
| **CENPB** |
| **CDC25B** |
| **RASSF2** |
| **MCM8** |
| **BMP2** |
| **KAT14** |
| **CST1** |
| **TPX2** |
| **POFUT1** |
| **KIF3B** |
| **NOL4L** |
| **E2F1** |
| **TP53INP2** |
| **MMP24OS** |
| **NORAD** |
| **EPB41L1** |
| **MYL9** |
| **SOGA1** |
| **RBL1** |
| **VSTM2L** |
| **BPI** |
| **FAM83D** |
| **ZHX3** |
| **MYBL2** |
| **TOX2** |
| **FITM2** |
| **PIGT** |
| **UBE2C** |
| **MMP9** |
| **PREX1** |
| **TMEM189** |
| **PTPN1** |
| **PARD6B** |
| **NFATC2** |
| **AURKA** |
| **CASS4** |
| **RBM38** |
| **APCDD1L** |
| **LINC01711** |
| **NPEPL1** |
| **C20orf197** |
| **CABLES2** |

| |
|---|
| NTSR1 |
| SLC17A9 |
| PPDPF |
| SAMD10 |
| HCN2 |
| DAZAP1 |
| TCF3 |
| AP3D1 |
| SF3A2 |
| LMNB2 |
| ZNF556 |
| TLE2 |
| GNA11 |
| TBXA2R |
| ZBTB7A |
| ANKRD24 |
| CHAF1A |
| TNFAIP8L1 |
| UHRF1 |
| PTPRS |
| KHSRP |
| SLC25A23 |
| TNFSF14 |
| C3 |
| PNPLA6 |
| CTXN1 |
| ELAVL1 |
| RAB11B |
| PRAM1 |
| COL5A3 |
| DNMT1 |
| MRPL4 |
| ICAM5 |
| RAVER1 |
| TYK2 |
| S1PR5 |
| KRI1 |
| ILF3 |
| CARM1 |
| SMARCA4 |
| LDLR |
| SPC24 |
| KANK2 |
| DOCK6 |
| RAB3D |

| |
|---|
| PLPPR2 |
| PRKCSH |
| MAN2B1 |
| RAD23A |
| NFIX |
| PODNL1 |
| SAMD1 |
| PRKACA |
| ASF1B |
| ADGRL1 |
| TECR |
| ADGRE3 |
| ADGRE2 |
| BRD4 |
| KLF2 |
| CHERP |
| SLC35E1 |
| SIN3B |
| CPAMD8 |
| ANKLE1 |
| GTPBP3 |
| LOC100507551 |
| COLGALT1 |
| UNC13A |
| FCHO1 |
| RAB3A |
| SSBP4 |
| KXD1 |
| CRTC1 |
| NUDT19 |
| KCTD15 |
| GRAMD1A |
| HAUS5 |
| PROSER3 |
| WDR62 |
| SPRED3 |
| ACTN4 |
| LTBP4 |
| NUMBL |
| C19orf54 |
| CYP2S1 |
| HNRNPUL1 |
| ATP1A3 |
| ZNF574 |
| POU2F2 |

| |
|---|
| GSK3A |
| LIPE |
| BCAM |
| ERCC2 |
| PPP1R13L |
| CD3EAP |
| BHMG1 |
| SIX5 |
| DMPK |
| DMWD |
| MYPOP |
| STRN4 |
| NPAS1 |
| BICRA |
| EHD2 |
| TMEM143 |
| KDELR1 |
| GYS1 |
| SNRNP70 |
| PPFIA3 |
| PRR12 |
| AKT1S1 |
| KCNC3 |
| SIGLEC9 |
| SIGLEC7 |
| SIGLEC17P |
| CLDND2 |
| NLRP12 |
| MYADM |
| OSCAR |
| CNOT3 |
| MBOAT7 |
| LENG8 |
| LILRA1 |
| FCAR |
| PPP6R1 |
| KMT5C |
| IL11 |
| ZNF628 |
| SSC5D |
| ZNF865 |
| ZNF580 |
| CCDC106 |
| ZNF814 |
| ZNF8 |

| |
|---|
| ZBTB45 |
| TRIM28 |
| PEX26 |
| LOC102725072 |
| RTL10 |
| ZDHHC8 |
| PPM1F |
| GNAZ |
| BCR |
| SNRPD3 |
| GRK3 |
| PITPNB |
| ZNRF3 |
| KREMEN1 |
| GAS2L1 |
| LIF |
| LOC91370 |
| SF3A1 |
| SLC35E4 |
| OSBP2 |
| MORC2 |
| SMTN |
| YWHAH |
| TIMP3 |
| RASD2 |
| RBFOX2 |
| IL2RB |
| C1QTNF6 |
| RAC2 |
| ELFN2 |
| CDC42EP1 |
| SH3BP1 |
| PLA2G6 |
| CBX7 |
| XPNPEP3 |
| ZC3H7B |
| SREBF2 |
| CYB5R3 |
| MCAT |
| TTLL12 |
| SHISAL1 |
| RTL6 |
| GTSE1 |
| CELSR1 |
| GRAMD4 |

| |
|---|
| CERK |
| ADM2 |
| SHANK3 |
| KCNE1B |
| CXADR |
| JAM2 |
| URB1 |
| LINC00649 |
| SLC5A3 |
| CHAF1B |
| TTC3 |
| RRP1B |
| UBE2G2 |
| COL18A1 |
| SLC19A1 |
| LINC01694 |
| COL6A1 |
| COL6A2 |
| SPATC1L |
| LSS |
| C21orf58 |
| PCNT |
| S100B |
